# Aluminum-Induced Surface-Enhanced Raman Scattering in Ti-Al-Ti Sandwich Multilayer Thin Films

**DOI:** 10.3390/nano16030216

**Published:** 2026-02-06

**Authors:** Luping Wu, Tingzhen Yan, Ruijin Hong, Chunxian Tao, Qi Wang, Hui Lin, Zhaoxia Han

**Affiliations:** 1 Engineering Research Center of Optical Instrument and System, Ministry of Education and Shanghai Key Lab of Modern Optical System, University of Shanghai for Science and Technology, No. 516 Jungong Road, Shanghai 200093, China; lp06345@usst.edu.cn (L.W.);; 2 Department of Printing and Pack Aging Engineering, Shanghai Publishing and Printing College, No. 100 Shuifeng Road, Shanghai 200093, China

**Keywords:** surface-enhanced Raman scattering, sandwich structure, optical thin films, titanium aluminum multilayer films

## Abstract

A series of Ti-Al-Ti sandwich thin films with different Al layer thicknesses was prepared via magnetron sputtering. The Al layer facilitated Ti-Al metal coupling within the films, which significantly strengthened the localized surface plasmon resonance (LSPR) and obtained more “hot-spots”, ultimately leading to a remarkable enhancement of the localized electric field. The LSPR effectively promoted charge transfer between probe molecules and the Ti-Al-Ti sandwich thin film. Raman scattering intensity was jointly governed by chemical and electromagnetic enhancement mechanisms. When used as a surface-enhanced Raman scattering (SERS) substrate for methylene blue (MB) detection, the sandwich-structured films achieved a Raman enhancement factor of 3.27 × 10^6^, approximately twice that of single-layer silver thin films. The substrate exhibited a low MB detection limit for MB of 10^−8^ M and excellent stability. Additionally, the relative standard deviation of main characteristic peak intensities across different positions is consistently below 6%, indicating superior uniformity and reproducibility. Experimental results are in good agreement with FDTD simulation outcomes.

## 1. Introduction

Raman scattering is an inelastic scattering phenomenon that occurs after the interaction between light and molecules, and the signal is usually weak and difficult to detect. Surface enhancement of Raman scattering (SERS) can enhance the Raman signal of target molecules by millions or even billions of times by triggering strong local electromagnetic fields on the surface of metal nanostructures, thereby achieving ultra-high sensitivity detection of molecules and materials utilized extensively in fields such as biomedical testing, materials science, environmental monitoring, food safety, and chemistry [1,2,3,4,5,6,7,8].

The core of SERS substrate material is to generate strong local electromagnetic fields through the special structure of the metal surface, significantly enhancing the Raman scattering signal of the target molecule. Among them, gold (Au) and silver (Ag) nanoparticles have become the most commonly used SERS substrate materials due to their excellent surface plasmon resonance properties. Researchers optimize the surface plasmon resonance effect of nanoparticles by precisely controlling their shape, size, and spacing, thereby significantly enhancing Raman signals [9,10,11,12,13,14].

In addition to noble metal nanoparticles, composite materials and hybrid structures have also become hot topics in the research of SERS substrate materials. Metal semiconductor composite materials (such as gold titanium dioxide nanocomposites) exhibit excellent SERS performance by combining the plasmon resonance effect of metal nanoparticles and the photocatalytic properties of semiconductor materials. This type of composite material not only enhances Raman signals but also degrades organic pollutants through photocatalysis, with multifunctional application potential [15,16]. Hybrid structures such as metal–organic frameworks (MOFs) combined with metal nanoparticles utilize the highly ordered and porous structure of MOFs, as well as the enhancement effect of metal nanoparticles, to construct efficient SERS substrates and achieve high-sensitivity detection of complex samples [17,18]. Some precious metal oxides, as Raman substrate materials, have disadvantages in surface-enhanced Raman scattering technology, such as poor chemical stability, complex “hotspot” construction, and poor biocompatibility [19,20].

To overcome the limitations imposed by materials, researchers have gradually shifted their focus from noble metal substrates to substitutes like non-noble metals and even non-metallic materials. Recent notable advancements have been made in exploring two-dimensional materials (e.g., graphene and transition metal disulfides) as SERS substrates [21]. Graphene is considered an ideal SERS substrate material due to its unique electronic structure and excellent conductivity. Research has shown that graphene can not only significantly enhance Raman signals but also interact strongly with target molecules through its π-π stacking effect, thereby further improving detection sensitivity [22]. Similarly, the layered structure and surface plasmon resonance effect of transition metal disulfides (such as MoS_2_) demonstrated significant application value in biosensing and environmental monitoring [23].

In many applications, SERS substrates require high reproducibility and long-term stability. Ordered noble metal nanostructures can provide uniform hotspot distribution, thus exhibiting good signal reproducibility and uniformity as SERS substrates. The most commonly used methods for preparing ordered noble metal nanoarrays include interface self-assembly [24], the template method [25], and the micro-nano processing method [26]. Micro-nanofabrication is an important method for preparing ordered metal nanoarrays, including electron beam lithography [27], focused ion beam lithography [28], nanoimprint lithography [29], reactive ion etching [30], laser interference lithography [31], nanosphere lithography [32], etc. Combined with thin film deposition technology, morphology and structure can be designed on demand at a more precise scale. However, the main drawbacks of micro-nano processing technology in preparing surface-enhanced Raman scattering (SERS) substrates are the contradiction between processing accuracy and cost, insufficient structural stability and repeatability, material and process compatibility limitations, thermal mismatch issues, abnormal filling of melt, difficulty in large-scale production, and complexity of functional integration. It remained challenging to industrialize these materials and promote their application. How to obtain SERS substrate materials with high sensitivity, selectivity, repeatability, and large-scale production is the future Raman scattering technology research direction. Table 1 summarizes the SERS of different Ti or Ag composite material structures.

In this study, we introduce a cost-efficient method for the nanofabrication of effective large-area SERS substrates through a straightforward fabrication process. Multilayer films consisting of Ti-Al-Ti were produced using magnetron sputtering at ambient temperature. This paper also examines how the thickness of the Al layer affects the structural, optical, and SERS characteristics of the multilayer thin films. To analyze the electronic field distribution of these thin films with different Al layer thicknesses, the finite-difference time domain (FDTD) method was utilized.

## 2. Materials and Methods

**Figure 1:**
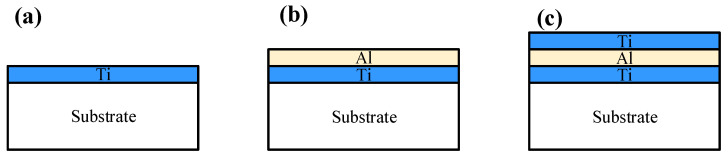
Structures of (**a**) Ti single-layer thin film, (**b**) Ti-Al bilayer thin film, and (**c**) Ti-Al-Ti multilayer thin film.

Ti-Al-Ti multilayer films were fabricated on fused quartz substrates through magnetron sputtering with titanium (99.9%) and aluminum (99.9%) targets, respectively. The deposition chamber was evacuated to a base pressure less than 3.0 × 10^−4^ Pa. Film growth was carried out in high-purity argon as working gas and at a constant working pressure of 0.8 Pa with the deposition rate of 1 nm/min. A titanium thin film, measuring 20 nm in thickness, was initially deposited onto the surface of a quartz substrate. Subsequently, the aluminum target materials were substituted, and under the identical vacuum conditions, an aluminum film with a thickness ranging from 5 to 20 nm was deposited onto the titanium thin films. Finally, a top layer of titanium thin film, also 20 nm thick, was applied, resulting in the formation of a Ti-Al-Ti sandwich structure. We named the Ti-Al-Ti sandwich structure samples with the Al layer thickness of 5 nm, 10 nm, 15 nm, and 20 nm as sample 1 (S1), sample 2 (S2), sample 3 (S3), and sample 4 (S4), respectively. For comparison, titanium, aluminum single-layer films, and Ti-Al bilayer metal films (each with a uniform thickness of 20 nm) were also fabricated. The thickness of each layer was precisely controlled and monitored via an in situ quartz crystal microbalance. To gain a better understanding of the sample structure, a schematic of the experimentation is shown in Figure 1.

The phases of the samples were characterized by X-ray diffraction (XRD, Rigaku MiniFlex 600 system, Tokyo, Japan). The surface roughness of samples was measured by atomic force microscopy (AFM, XE-100, Park System, Suwon, Republic of Korea). The optical absorption of the samples was measured by a UV-vis-NIR double-beam spectrophotometer (Lambda 1050, Perkins Elmer, Waltham, MA, USA). Additionally, the spectra of Raman scattering were obtained using a confocal microprobe Raman system (inVia Raman Microscope, Renishaw, UK) that operated at a wavelength of 633 nm, with the laser power set to 3 mW. All measurements took place at room temperature.

## 3. Results and Discussion

**Figure 2:**
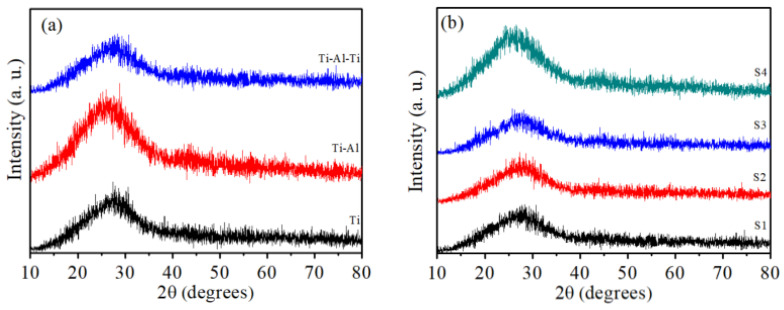
XRD patterns of (**a**) Ti single-layer thin film, Ti-Al bilayer thin film, and Ti-Al-Ti multilayer thin film samples, and (**b**) Ti-Al-Ti multilayer thin film with different Al layer thickness.

The XRD patterns of samples comprising a single-layer metal thin film, as well as bilayer and multilayer thin films, are illustrated in Figure 2. From the seen Figure 2a, it is evident that whether it is a single-layer metal titanium film, a metal Ti-Al bilayer film, or a metal Ti-Al-Ti multilayer film, they all exhibit an amorphous structure, and the metal Ti-Al-Ti multilayer film sample does not change its crystalline state with the increase in aluminum layer thickness, as shown in Figure 2b. This is mainly due to the thin thickness of each film layer, which may lead to poor crystallinity, so there are no pronounced diffraction signals of either metal, Ti, or Al thin films.

**Figure 3:**
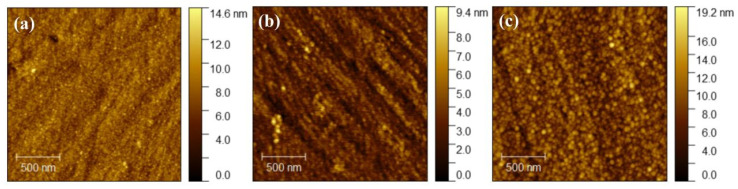
Atomic force microscopy images of (**a**) Ti single-layer thin film, (**b**) Ti-Al bilayer thin film, and (**c**) Ti-Al-Ti multilayer thin film with sandwich structure.

Figure 3 shows AFM images of several representative samples. From the figure, it can be seen that as the film structure transitions from a single layer to bilayer and multiple layers, the surface roughness shows a regular change. Due to the columnar crystal growth characteristics, the single-layer titanium film sample has a smooth surface and relatively low roughness, with a root mean square roughness value of only 1.193 nm. For the Ti-Al bilayer sample, its surface remains smooth, and the cover layer of the aluminum film does not increase its surface roughness, with a root mean square roughness (RMS) value of 0.9884 nm. This is because when an aluminum intermediate layer is introduced to form a Ti-Al bilayer structure, the aluminum layer can effectively fill the grain boundaries of the titanium layer. While for the Ti-Al-Ti composite film with sandwich structure, compared with the single-layer and double-layer film samples, the surface becomes significantly rougher, and the root mean square roughness value increases to 2.217 nm. This is because in the Ti-Al-Ti sandwich structure, the secondary deposition of the top titanium layer will trigger new grain competition growth. This change pattern is closely related to the interface diffusion effect and stress redistribution mechanism, among which the smoothing effect of the aluminum layer and the surface reconstruction effect of the titanium layer jointly dominate the morphological evolution of the multilayer system.

**Figure 4:**
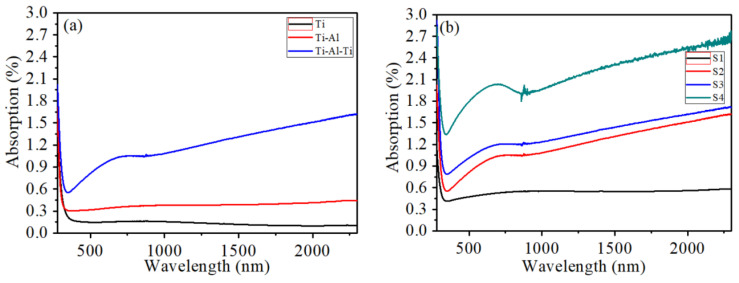
The absorption spectra of (**a**) Ti single-layer thin film, Ti-Al bilayer thin film, and Ti-Al-Ti multilayer thin film samples, and (**b**) Ti-Al-Ti multilayer thin film with different Al layer thicknesses.

Figure 4 illustrates the optical absorption curves of single-layer metal thin film, bilayer, and multi-layer thin film samples. From Figure 4a, it can be seen that due to the scattering effect of free electrons on photons, the overall absorption of the single-layer titanium film remains at a certain value, with a wide absorption band in the 500–1500 nm range, which is related to the band transition absorption edge of titanium. Compared with the single-layer titanium film, the absorption curve of the Ti-Al bimetallic film sample shows a significant bimetallic coupling effect, with aluminum’s free electrons dominating and the overall absorption rate increasing. However, no significant plasmon absorption peak was detected. In the case of Ti-Al-Ti alloy thin film samples with a sandwich structure, compared with single-layer and double-layer thin film samples, their overall absorption intensity is obviously increased, and there is a plasmon absorption peak in the visible light region. Due to lattice mismatch and atomic diffusion, a transition layer is formed at the interface of Ti-Al-Ti multi-layer films, which enhances electron localization and may trigger localized surface plasmon resonance. With the thickness of the Al layer increasing, the absorption intensity of the sandwich structure thin film sample increases correspondingly, and the absorption peak in the visible light region becomes more pronounced, indicating that variations in the thickness of the aluminum layer significantly regulate the plasmon coupling strength.

**Figure 5:**
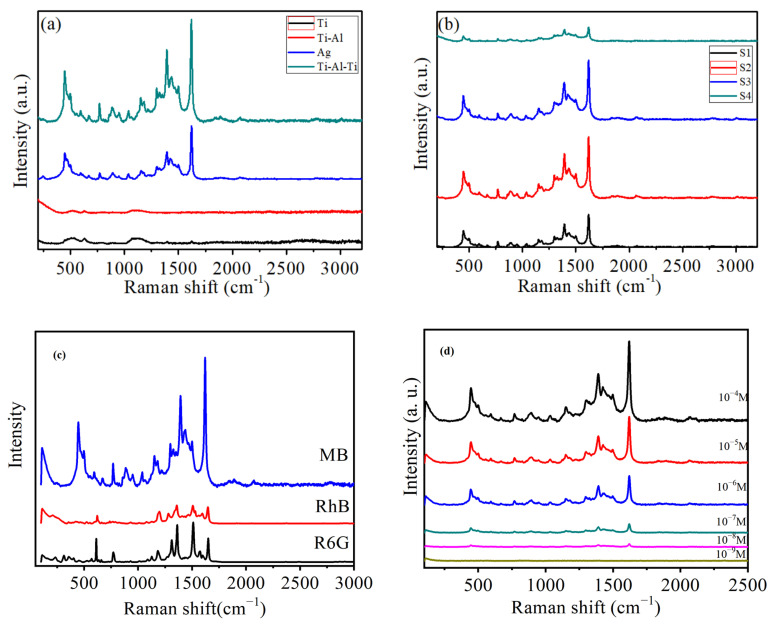
(**a**) The Raman spectra of MB (10^−4^ M) on Ti thin film, Ti-Al bilayer thin film, Ag thin film, and Ti-Al-Ti multilayer thin film with sandwich structure, (**b**) presents SERS spectra obtained from substrates with varying Al layer thicknesses, and (**c**) Raman spectra of different probe molecules. (**d**) The Raman spectra of MB at different analyte concentrations.

To further verify the electric field enhancement and potential applications of the Ti-Al-Ti sandwich-structured multilayer metal film, Raman spectroscopy was adopted to examine the SERS performance of single-layer Ti films, Ti-Al bimetallic films, and Ti-Al-Ti sandwich multilayer films with varying Al layer thicknesses. Methylene blue (MB) was used as the SERS probe molecule at a concentration of 1 × 10^−4^ mol/L, and the Raman spectroscopy was operated with a 633 nm excitation wavelength; the test results are presented in Figure 5. As shown in Figure 5a, MB molecules are barely detectable on the single-layer Ti film and Ti-Al bimetallic films. In contrast, the Ti-Al-Ti sandwich-structured multilayer films exhibit distinct Raman characteristic peaks of MB, which significantly enhance the detection signal intensity of MB molecules. This indicates that under the excitation of 633 nm excitation light, the sandwich structure Ti-Al-Ti multilayer metal film exhibits better a SERS effect compared to either a single-layer titanium metal film or a titanium aluminum bilayer composite film. The figure clearly illustrates that the Ti-Al-Ti multilayer metal film, which has a sandwich structure, exhibits distinctive peaks at 447 cm^−1^, 1394 cm^−1^, and 1621 cm^−1^. These peaks correspond to the C-N-C vibration modes, symmetric stretching vibration of the C-N bond, and cyclic stretching vibration of the C-C bond, respectively [39].

The Raman signal intensity of MB molecules rose as the Al layer thickness increased up to 15 nm, then dropped sharply when the Al layer thickness was further increased, as illustrated in Figure 5b. The Raman spectra results of the Ti/Al/Ti sandwich structure in Figure 5a are consistent with those of the S3 sample in Figure 5b, with a thickness of 55 nm. In the sandwich structure, the electromagnetic field is confined to the middle aluminum layer, and the coupling effect between the titanium metal and aluminum metal thin film generates a localized surface plasmon absorption effect. With the thickness of the Al layer increasing, the local surface plasmon absorption intensity of the sandwich-structured thin film sample correspondingly increases. Due to a certain relationship between Raman scattering intensity and substrate material thickness, but not a simple nonlinear relationship. In general, as the thickness of the substrate material increases, the Raman scattering intensity will also increase. However, as the thickness of the substrate material further increases, the Raman scattering intensity will decrease. This is because an increase in thickness may lead to insufficient laser penetration or absorption of scattered light intensity.

To verify the universality of this substrate material, we used three probe molecules, namely rhodamine B, R6G, and methylene blue, for the verification. The results showed that this base material had the best detection effect on the methylene blue probe molecule, as shown in Figure 5c. Figure 5d presents the SERS performance of the S3 sample for different MB concentrations (ranging from 10^−4^ to 10^−9^ mol/L). The lowest detectable concentration of MB is 10^−8^ mol/L.

**Figure 6:**
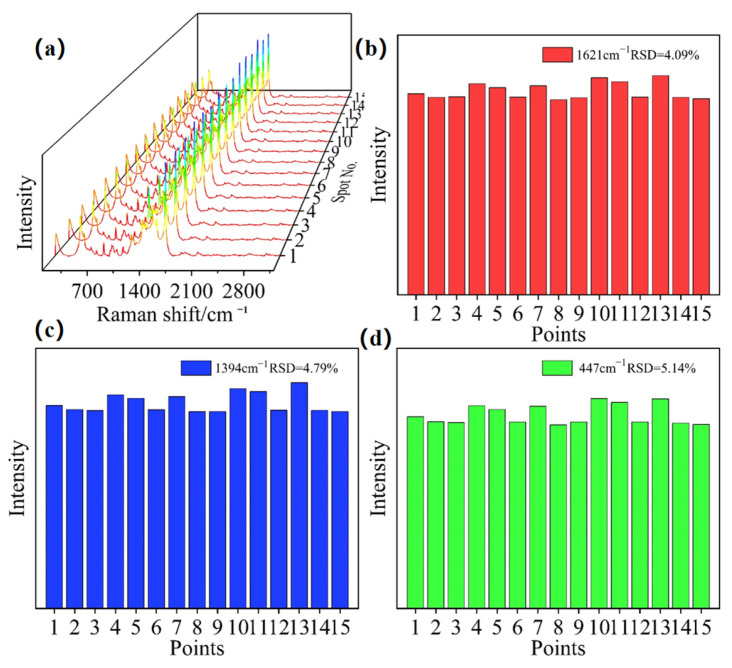
(**a**) SERS spectra collected from different spatial locations, and (**b**–**d**) the corresponding RSD values of the characteristic peaks at 1623 cm^−1^, 1393 cm^−1^, and 447 cm^−1^, respectively.

An optimal SERS substrate must exhibit high detection sensitivity. To explore the detection sensitivity of the sample, we selected a sandwich structure for a more in-depth analysis of its SERS capabilities. Figure 6a shows SERS spectra collected from different spatial locations. A gradual reduction in the detected Raman signal intensity is noted as the MB concentration decreases. At a concentration of 10^−8^ M, only faint Raman signals are observable. Furthermore, no MB related characteristic peaks are detectable at a concentration of 10^−9^ M, indicating the sample’s detection limit is 10^−8^ mol/L. The enhancement factor (*EF*) is the most direct metric for Raman enhancement efficacy; thus, the *EF* of the sandwich-structured sample at 1623 cm^−1^ was calculated via the given formula [40]:

(1) $$EF=\frac{I_{sers}}{I_{bulk}}\times \frac{C_{bulk}}{C_{sers}}$$

Here, *I_sers_* represents the Raman characteristic peak intensity of the target molecule on the SERS substrate, while *I_bulk_* refers to the Raman signal intensity of the same molecule without SERS enhancement, measured under the same vibrational mode. The variables *C_sers_* and *C_bulk_* correspond to the concentrations of the detected molecule with and without SERS substrate enhancement, respectively. Calculations show that the enhancement factors (*EF*) of the single-layer silver film at 1394 cm^−1^ and 1621 cm^−1^ are 1.34 × 10^6^ and 1.80 × 10^6^, respectively. For the Ti-Al-Ti sandwich-structured multilayer film, the EFs at the same wavenumbers are 2.50 × 10^6^ and 3.27 × 10^6^, nearly doubling those of the single-layer silver film.

To better characterize the SERS uniformity of the sample, the Ti-Al-Ti sandwich-structured multilayer film was coated with a large-area drop of 1 × 10^−4^ mol/L MB dye. Subsequently, 15 random points on the sample surface were selected for Raman measurement. The relative standard deviation (RSD) values of the MB characteristic peak intensities at 447 cm^−1^, 1393 cm^−1^, and 1623 cm^−1^ were calculated, as shown in Figure 6b–d. The results revealed that all RSD values were below 6%, demonstrating excellent large-area uniformity of the prepared SERS substrate.

**Figure 7:**
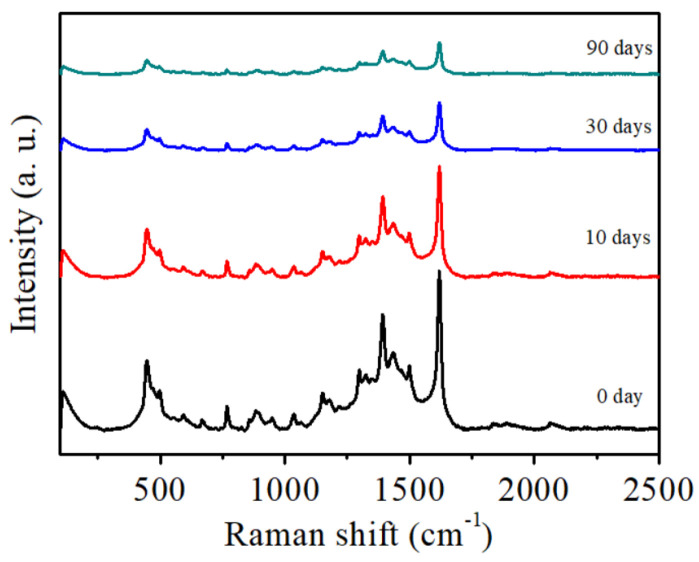
The Raman spectra of MB (10^−4^ M) on the Ti-Al-Ti multilayer thin film with a sandwich structure stored for different periods of time.

Substrate stability plays a vital role in practical applications. To assess the stability of the samples, same-batch specimens were stored in a natural environment for a specified duration and periodically tested for the Raman spectra of methylene blue (MB) molecules. Figure 7 presents the time-dependent Raman spectra of the air-exposed samples. It shows that within the initial 15 days, the Raman scattering intensity decreased compared with that of the freshly prepared sample. By the 90th day, the MB Raman signal intensity decreased significantly; however, the MB characteristic peak remained detectable with negligible peak position shift. This is because the gradual oxidation of surface Ti/Al layers, which covers the critical “hot spot” regions (nanogaps and tips) and reduces the effective metallic area for localized surface plasmon resonance (LSPR). Notably, the detectable MB characteristic peaks with negligible shifts after 90 days confirm the absence of significant MB chemical degradation or substrate nanostructure collapse. The results indicate that the Ti-Al-Ti sandwich-structured multilayer film exhibits good stability as an SERS substrate.

**Figure 8:**
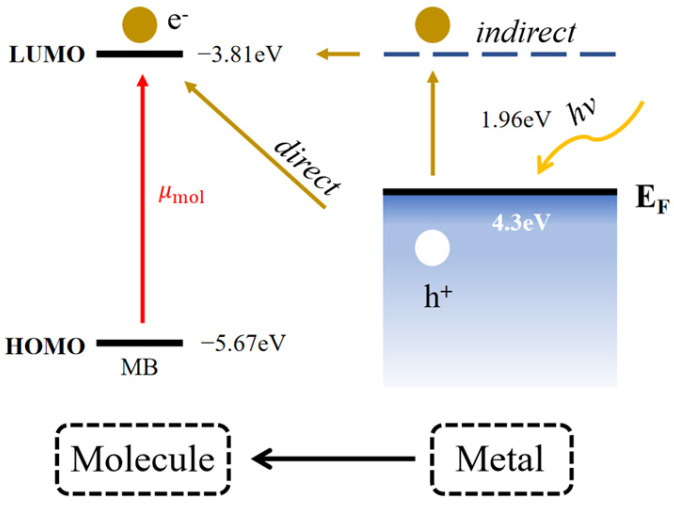
Band maps of charge transfer pathways in Ti-Al-Ti multilayer thin film with sandwich structure and MB.

The improved SERS mechanism typically arises from the synergistic effects of chemical enhancement (CM) and electromagnetic enhancement (EM). A schematic illustration depicting the augmented photoinduced charge transfer between the Ti-Al-Ti multilayer metal film and the adsorbed MB probe molecules is presented in Figure 8. Previous studies have shown that the highest occupied molecular orbital (HOMO) and lowest unoccupied molecular orbital (LUMO) energies of MB are −5.67 eV and −3.81 eV, respectively [41]. Quantitative verification: ΔE (E-|E|) = 4.30 − 3.81 = 0.49 eV. Since hν (1.96 eV) ≫ ΔE, electron transfer from the substrate to the MB LUMO is feasible, confirming the charge transfer mechanism. This also supports μ_PICT_ dominance: Ti-Al coupling induces LSPR, reducing charge transfer barriers and synergizing with EM to enhance Raman signals, consistent with our results. Under laser excitation, the system undergoes resonances and charge transfers driven by strong interfacial interactions between MB and the SERS substrate, thus triggering the chemical enhancement mechanism. According to Figure 8, upon laser irradiation, adsorbed intramolecular electrons are usually directly excited from HOMO to LUMO, resulting in a resonance Raman scattering effect. The photo-driven molecule-metal interaction generates new charge transfer resonance states, forming a charge transfer enhancement mechanism. According to the direction of charge transfer, charge transfer can be divided into metal-to-molecule. According to the charge transfer pathway, the mechanism of charge transfer enhancement can usually be divided into two types: direct and indirect charge transfer Direct charge transfer enhancement refers to the direct transition of electrons from a metal to a molecular LUMO (or from a molecular HOMO to an orbital above the metal Fermi level) under photoexcitation Indirect charge transfer enhancement is the generation of hot charge carriers under photoexcitation, which undergo charge transfer through resonance tunneling. Hot electrons transfer to the LUMO of the molecule, while hot holes transfer to the HOMO of the molecule.

Among them, μPICT plays a dominant role, and the mechanism occurs mainly due to the coupling between titanium metal and aluminum metal. A plasmon resonance absorption peak was generated in the visible light band, and the absorption intensity varies with the thickness of the aluminum layer. The existence of plasmon resonance absorption enhances electron transfer to the molecule more efficiently. It is evident that localized surface plasmon resonance significantly boosts charge transfer efficiency, thereby increasing the intensity of the Raman signal.

**Figure 9:**
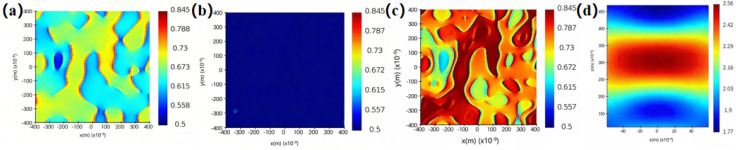
FDTD patterns of (**a**) Ti single-layer thin film, (**b**) Ti-Al bilayer thin film, and (**c**) Ti-Al-Ti multilayer thin film with sandwich structure, and (**d**) z-axis electric field of Ti-Al-Ti three-layer sandwich-structured thin film.

To further explore the correlation between Raman enhancement and electromagnetic enhancement in ablated thin film samples, the local electric field distribution of the samples was calculated via the Finite Difference Time Domain (FDTD) method before and after laser irradiation. In this simulation, a plane wave was adopted as the light source (excitation wavelength: 633 nm), incident normally on the sample’s x-y plane along the z-axis. Additionally, periodic boundary conditions were employed, the grid division accuracy was fixed at 1.5 nm, and an x-y plane monitor was configured to observe the system’s local electric field distribution. The electric field distributions of the single-layer Ti film and Ti-Al bilayer composite film are depicted in Figure 9a,b, respectively. Figure 9c illustrates the surface electric field distribution of the Ti-Al-Ti three-layer sandwich composite film, with its z-axis electric field distribution given in Figure 9d. For the single-layer Ti film and Ti-Al bilayer composite film, the overall electric field distribution is comparatively weak due to their dense and uniform surfaces. However, for Ti-Al-Ti three-layer composite thin film samples, the electromagnetic field in the sandwich structure is confined to the middle aluminum layer, resulting in a localized surface plasmon resonance effect. It is worth noting that the electric field is significantly enhanced at the interfaces of the smooth metal films in our simulation system, which can be primarily attributed to the excitation of propagating surface plasmon resonance (SPR) on the smooth metal surfaces. This prominent electric field enhancement originates from the coupling effect of surface plasmons at the interfaces between different metal layers, which enables the effective confinement of light in a small spatial volume and thus realizes the localized enhancement of the electric field. The results indicate that the local electric field of the Ti-Al-Ti three-layer composite film sample is significantly stronger than that of the single-layer metal Ti film and Ti-Al bilayer metal composite film samples, which is consistent with the experimental results.

## 4. Conclusions

This study introduces an economical and straightforward technique for creating sandwich structures intended for SERS substrates. The sandwich-structured samples were produced via the magnetron sputtering method at ambient temperatures. Verification through Raman scattering spectroscopy utilizing methylene blue as a probe revealed that the Ti-Al-Ti sandwich configuration effectively enhances SERS intensity. The multilayer thin film featuring the Ti-Al-Ti sandwich structure demonstrates commendable uniformity and stability, presenting benefits like a brief production cycle, affordability, and compatibility with large-scale manufacturing. Additionally, it offers a viable solution for the detection of trace molecules in practical applications.

## Figures and Tables

**Table 1:** SERs of different Ti or Ag composite material structures.

Material System	Probe Molecule	Detection Concentration	Enhancement Factor	Stability/Reproducibility	Ref.
Ag/TiO_2_ nanotubes/Ti	Pyridine	0.05 M	Higher than Ag reference (Ag ≥ 0.06 mg/cm^2^)	Air-stable; RSD ≤ 20%	[33]
4MBA/Ag/Ag-doped TiO_2_	4-Mercaptobenzoic acid (4MBA)	Not specified	1.68 × 10^6^	Irreversible accumulation; saturated after 80 s NIR	[34]
Ag-2%Ti alloy nanorods	Methylene blue (MB)	5 × 10^−9^ M–1 × 10^−5^ M	~40% of pure Ag NRs	Stable in air (≥35 days) and 10 mM HNO3 (1 h); RSD = 4.86%	[35]
Ti−AgNRs@Al_2_O_3_	Methylene blue (MB)	1 × 10^−6^ M	Comparable to pure AgNRs	Critical load = 5.40 mN; stable after 15 min ultrasonic vibration	[36]
Ag/TiO_2_/Ti foam	Rhodamine 6G (R6G)	2.24 × 10^−11^ M–1 × 10^−6^ M	1.9 × 10^7^	Air-stable (3 months); RSD = 8.4%	[37]
Ti/TiN/Ag	Methylene blue (MB)	1.5 × 10^−6^ M	37× higher than as-deposited Ti	Oxidation-resistant;	[38]

## Data Availability

The original contributions presented in this study are included in the article. Further inquiries can be directed to the corresponding author.
